# Health behaviours of Australian men and the likelihood of attending a dedicated men’s health service

**DOI:** 10.1186/s12889-018-5992-6

**Published:** 2018-08-30

**Authors:** Andrew D. Vincent, Phoebe G. Drioli-Phillips, Jana Le, Lynette Cusack, Timothy J. Schultz, Margaret A. McGee, Deborah A. Turnbull, Gary A. Wittert

**Affiliations:** 1 Freemasons Foundation Centre for Men’s Health, Adelaide Medical School, Faculty of Health and Medical Sciences, The University of Adelaide, Adelaide, South Australia 5005; 2 School of Psychology, Faculty of Health and Medical Sciences, The University of Adelaide, Adelaide, South Australia 5005; 3 Adelaide Nursing School, Faculty of Health and Medical Sciences, The University of Adelaide, Adelaide, South Australia 5005

**Keywords:** Health services, men’s health, Health help-seeking, Health behaviours

## Abstract

**Background:**

Redesigning primary health services may enhance timely and effective uptake by men. The primary aim of this study was to assess the likelihood of Australian men attending a dedicated men’s health service (DMHS). The further aims were to better understand the reasons for their preferences and determine how health behaviours influence likelihood.

**Methods:**

A survey on health service use and preferences, health help-seeking behaviours, and the likelihood of attending a DMHS was administered by telephone to 1506 randomly selected men (median age 56 years, range 19–95). Likelihood of attending a DMHS was rated using a single item Likert scale where 0 was not at all likely and 10 highly likely. Respondents were classified by age (< or > = 65 years) and health status. Principal component analyses were used to define health behaviours, specifically help-seeking and delay/avoidance regarding visiting a doctor. Multivariable linear and logistic regression analyses were used to examine predictors of likelihood of attending a DMHS.

**Results:**

The mean likelihood of attending a DMHS was 5.8 (SD 3.3, median 6, moderate likelihood) and 21%, 26% and 23% of men rated likelihood as moderate, high and very high respectively. Being happy with their existing doctor was the most common reason (52%) for being less likely to attend a DMHS. In unadjusted analyses, younger men reported being more likely to attend a DMHS (*p* < 0.001) with older-sick men reporting being least likely (*p* < 0.001). Younger men were more likely than older men to score higher on delay/avoidance and were more likely to self-monitor. In the full model, men with current health concerns (*p* ≤ 0.01), who scored higher on delay/avoidance (*p* ≤ 0.0006), who were more likely to be information-seekers (*p* < 0.0001) and/or were motivated to change their health (*p* ≤ 0.0001) reported a higher likelihood of attending a DMHS irrespective of age and health status.

**Conclusions:**

Seventy percent of men reported a moderate or higher likelihood of attending a DMHS. As young healthy men are more likely than older men to display health behaviours that are associated with a higher likelihood of attending a DHMS, such as delay/avoidance, marketing a DMHS to such men may be of value.

**Electronic supplementary material:**

The online version of this article (10.1186/s12889-018-5992-6) contains supplementary material, which is available to authorized users.

## Background

There is a significant burden of undetected or poorly managed disease among Australian men that translates into males having, on average, nearly 5 years less equivalent ‘healthy life’ lived than females [1]. The susceptibility and response to disease differs between men and women due to inherent biological differences, sociocultural, economic, environmental and political influences [2]. Additionally, attitudes towards health and health service utilisation differ [3–8]. In particular, men are more likely to self-monitor their health status for longer and seek information independently prior to attending a health service [7]. When attending their general practitioner (GP), they have shorter consultations, see the GP later during their illness, leave significant health issues unattended, and are more likely to somatise emotional problems when compared to women [9, 10]. We have previously reported that there are core qualities that Australian men value when communicating with GPs [11], and yet health services rarely tailor their approach to these preferences. Consequently, men’s health issues may either go untreated or single problems are treated in isolation, leaving general health risk factors unassessed. Redesigning health services, including sex-specific health services, and the promotion of such health service use to men are strategies that may enhance timely and effective utilisation [12–14]. Promoting active health help-seeking and monitoring in younger men without established conditions is important for risk reduction and disease prevention. This is particularly important given that paternal health factors and lifestyle behaviours influence disease risk of subsequent generations [15].

While dedicated men’s health services in Australia exist, they are rare and published evidence to support this service delivery approach in terms of likelihood of use by men, and whether they can deliver better services and health outcomes beyond standard practice are lacking [3, 16]. The primary aim of this study was to assess the self-reported likelihood of Australian men attending a dedicated men’s health service (DMHS). Further aims were to gain a better understanding the reasons for their preferences and assess how health help-seeking behaviours and sociodemographic characteristics are associated with likelihood of attending a DMHS.

## Methods

Initially, a pilot study consisting of six focus group discussions involving 46 South Australian men aged 25–69 years was conducted. Discussions included their health help-seeking behaviours, the concept of a DMHS for men aged 18 years and older and the characteristics of a service considered important to men. The outcomes suggested an overall positive response to a proposed DMHS with a focus on comprehensive assessment and health management plans, permitting further exploration in a larger survey. Focus group and phone interviews were conducted by Interviewer Quality Control Australia (IQCA) accredited interviewers, in accordance with the Market & Social Research Privacy Principles. As market research, ethics approval and consent was waived by the Institutional Human Research Ethics Committee.

The primary outcome, likelihood of attending a DMHS, was assessed using a single item Likert scale. The question was phrased as follows: “The University … is considering establishing a men’s health service, staffed by professionals who are especially knowledgeable about men’s health. We want to know how appealing this idea is to men. Can you tell me, in principle, how likely you are to attend a dedicated men’s health service? Use a 0 to 10 scale where 0 is not at all likely to attend and 10 is highly likely to attend.” No details regarding the possible attributes of the service were provided before men answered this question. A priori, it was decided that respondents who rated likelihood as ≤ 5 out of 10 were asked to provide a main reason for being unlikely to attend a DMHS.

The survey included a further 71 questions regarding current health service utilisation and preferences, socio-demographics and health behaviours. The latter comprised 9 questions asking men to rate likelihood of health help-seeking behaviours for when they have health concerns or begin to experience symptoms of ill-health, and 13 questions containing statements on possible reasons for delay/avoidance behaviour regarding visits to their doctor for which men were asked to rate the extent to which they agree or disagree using a 0–10 scale (0 = strongly disagree, 10 = strongly agree).

The survey was administered by telephone interview to a random, non-replacement representative sample of adult men living in home residents across metropolitan and rural South Australia. Pre-survey advance letters were sent to a sample of 4900 addresses for numbers randomly drawn from the electronic telephone book. This initial sample size was based on the proportion of households with an adult male and in order to achieve the desired sample size of 1500 (margin for error with 95% confidence ±2.51). Upon answering, the caller asked to speak with the man in the household, aged 18 years or older, who had their birthday most recently. Allowing for households with no adult males, disconnected, inappropriate or unanswered numbers, and other ineligibility (non-english speaking, a disability preventing participation) there were 2344 responses (48%), and of these a participation rate of 64% (1506/2344) was achieved (Additional file 1: Table S1). The interviews were completed with the use of Computer Aided Telephone Interviewing technology. The mean interview time was 19.2 min.

Due to the phrasing of the primary question we refer to ‘likelihood’ throughout this article when stating the respondents’ likelihood of attending a DMHS, rather than the statistical interpretation of the word. Qualitative content analysis was performed to categorise the reasons given by men declaring a likelihood of attending a DMHS of <=5 into recurrent themes to identify the prominent elements [17, 18].

### Statistical methods

**Fig. 1:**
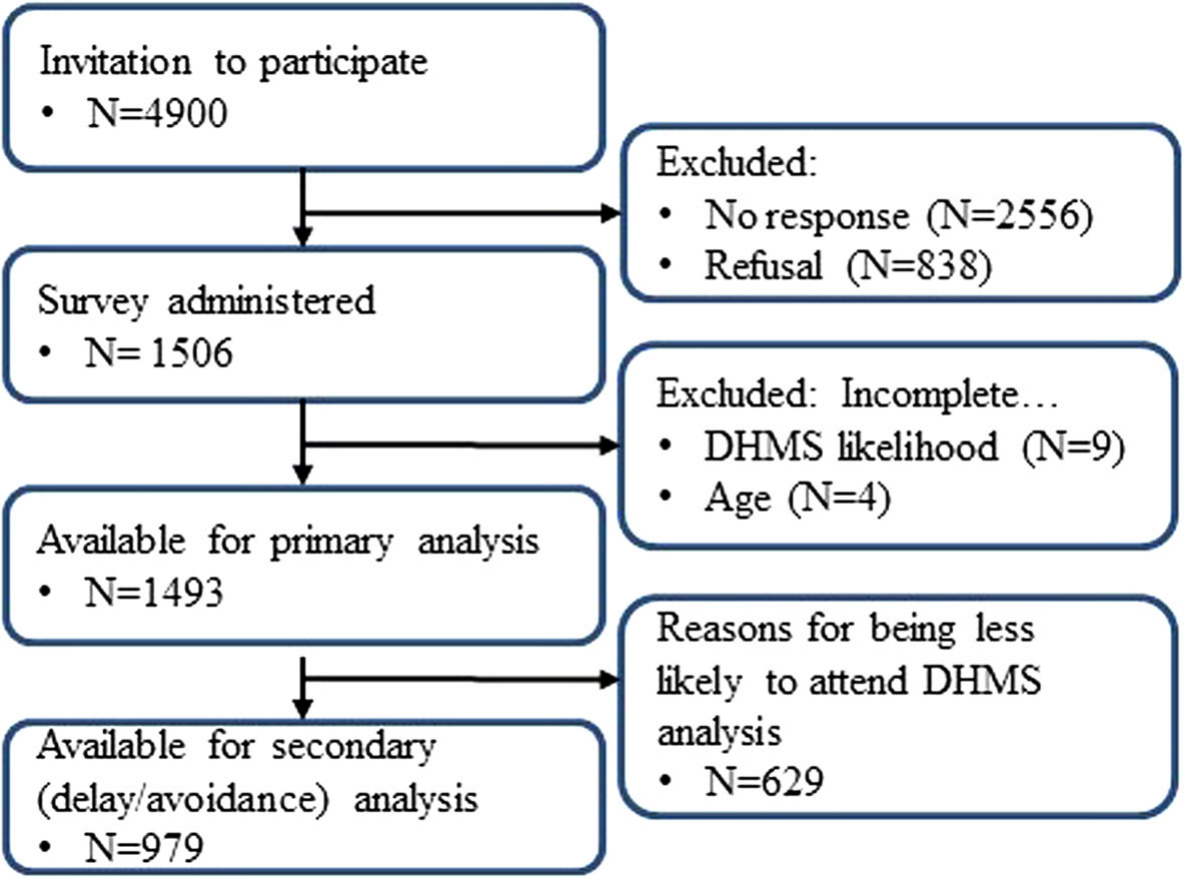
Study design

A priori we believed that age and health status would influence individual interest in attending a DMHS. Respondents were therefore categorised into four cohorts; young and healthy (YH), young and sick (YS), older and healthy (OH), and older and sick (OS), with the assumption that existing health service use would be different between these four groups. Health status was defined as “sick” if there was at least one chronic condition or cancer present (Fig. 1). A priori we believed that free-time availability would be a barrier to health service use, and as such age status was defined by being 65 years of age (the average intended retirement age of Australians) or older and/or retired. ANOVA F-tests were employed to compare summary statistics across the four discrete age-health groups for graphical summaries, otherwise we used generalized linear regressions with age as a continuous linear predictor, health status as dichotomous factor and included their pair-wise interaction when appropriate. Binomial logistic regression was used to assess associations between age and health status and delay/avoidance behaviour. Linear regressions were used in all other analyses.

Principal component analyses were employed to identify major axes of variation in the responses to the 9 help-seeking behaviours questions and 13 delay/avoidance behavior statements. The number of principal components were identified by visual inspection of scree plots. There were two major axes of variation (principal components) in the data from the 9 questions regarding help-seeking behaviours that explained 47% of the variance in the data (Additional file 1: Table S2). These components were categorised as “self-monitoring” and “information seeking” behaviours. There was only one main axis (principal component) in the delay/avoidance data explaining 33% of the variation in reasons for delaying/avoiding a doctor visit. An average delay/avoidance score was therefore derived from the 13 statements, which correlated almost perfectly with the first principal component (*r* = 0.99).

The final primary analyses of likelihood of attending  a DHMS was performed first in the entire cohort and secondly only in men reporting delay/avoidance behavior which allowed for the inclusion of a delay/avoidance score as a factor. Both analyses were repeated in the cohort of young healthy men without the health-status and age-health interaction terms. Responses from individuals not completing the primary likelihood question and/or missing age information were excluded. Other missing data was imputed using cohort means. A priori it was believed that health concerns, motivation to change, prior weight loss, age (younger/working v older/retired) and health status (healthy v sick) would affect a man’s response regarding the likelihood of attending a DMHS. As such, all these factors were included in the final analyses. Data analyses were conducted using R version 3.3.3 [19] (Fig. 1).

## Results

The survey was administered to 1506 men (Australian residents) of whom 9 did not respond regarding DHMS likelihood and 4 did not respond regarding age. The remaining cohort of 1493 had a median age of 56 years (mean 55.3 years, range 19–95 years) (Table 1). Sixty-five percent of respondents were in the work force and 40.3% of these were classified as blue collar workers, 27% were living in rural areas and 78% were married or in a de-facto relationship. Ten respondents (0.7%) were Aboriginal or Torres Strait Islander men. Ninety-two percent of all men (84% of young healthy men) had visited a doctor, either GP or specialist, at least once in the previous 12 months. This compares favourably to the national data of 76% of surveyed men who saw their GP at least once in the previous 12 months [20]. Seventy percent of all men and 69% of young healthy men had current concerns about their health, and most men (91%) reported being motivated to change their health (Additional file 1: Table S3).

**Table 1:** Likelihood of attending a DMHS by participant grouping

	*N* (%)	Mean Likelihood (SD)	*p*-value^a^
	1493 (100%)	5.8 (3.3)	
Likelihood			
0 (not at all likely)	203 (14%)		
1—4 (low)	237 (16%)		
5—6 (moderate)	313 (21%)		
7—8 (high)	391 (26%)		
9—10 (very high)	349 (23%)		
Age			
(18,34]	160 (11%)	5.9 (2.9)	< 0.0001
(35,49]	360 (24%)	6.2 (3.1)	
(50,64]	533 (36%)	6.1 (3.3)	
(65,79]	345 (23%)	5.2 (3.5)	
(80,95]	95 (6%)	5.1 (3.8)	
Region			
Rural	399 (27%)	6.0 (3.3)	0.22
Metro	1094 (73%)	5.7 (3.3)	
Employment status			
Employed (FT/PT)	971 (65%)	6.0 (3.2)	0.0002†
Not working/Student/Other	104 (7%)	5.9 (3.2)	
Retired	418 (28%)	5.2 (3.5)	
Occupation			
White collar	579 (60%)	6.0 (3.2)	0.53
Blue collar	391 (40%)	6.1 (3.4)	
Marital Status			
Divorced/Separated	91 (6%)	5.7 (3.3)	0.98
Married/Defacto	1165 (78%)	5.8 (3.3)	
Never married	189 (13%)	5.9 (3.2)	
Widowed	48 (3%)	5.7 (3.7)	
Household income			
< $50,000	531 (38%)	5.6 (3.5)	0.63
$50,000–$100,000	525 (37%)	6.2 (3.2)	
$100,000–$200,000	295 (21%)	5.8 (3.2)	
> $200,000	55 (4%)	5.3 (3.2)	
Health status			
Sick	567 (38%)	5.6 (3.5)	0.02
Healthy	926 (62%)	6.0 (3.2)	
Health-Age groups			
YH	733 (49%)	6.0 (3.1)	< 0.0001
YS	279 (19%)	6.2 (3.4)	
OH	193 (13%)	5.8 (3.5)	
OS	288 (19%)	4.9 (3.5)	
Number of Dr. visits (12 mths)			
Not at all	122 (8%)	5.6 (3.2)	0.73
Once or twice	428 (29%)	5.8 (3.3)	
3 to 5 times	428 (29%)	6.0 (3.2)	
5+ times	504 (34%)	5.7 (3.5)	

^a^linear regression associations (age as linear, income and Dr. visits as ordinal, others as nominal)

†*p* = 0.71 when excluding retirees (df = 1)

Given the distribution in responses (Table 1), likelihood of attending a DMHS was categorised as not at all likely (0), low (1–4), moderate (5–6), high (7–8) and very high (9–10) likelihood (Table 1). The mean likelihood rating for attending a DMHS for all men was 5.8 (moderate likelihood, SD 3.3, median 6, IQR 3–10). Seventy percent of men reported a likelihood of 5 or more.

In covariate unadjusted analyses, younger men (*p* < 0.0001) and men without chronic health conditions or cancer  (*p* = 0.02) were more likely to attend a DMHS, while older men with chronic health conditions or cancer were less likely to attend a DMHS (*p* < 0.001). Non-retired employment status, occupational status (white vs blue collar), marital status, household income, and frequency of attending the doctor were not associated with the likelihood rating.

Of the men reporting a likelihood rating of attending a DMHS of <=5, the three most common reasons given were that they were happy with their current general practitioner (*n* = 328; 52%), they were not interested in their health or felt that they could take care of themselves, not warranting a DMHS (*n* = 110 men; 17%), and possible time inconvenience in distance to travel (*n* = 99; 16%) (Table 2).

**Table 2:** Reasons given as to why respondents were unlikely to attend a DMHS

Themes	YH	YS	OH	OS	Total
	*N* = 292	*N* = 101	*N* = 83	*N* = 153	*N* = 629
Happy with current general practitioner	130 (45%)	54 (53%)	52 (63%)	92 (60%)	328 (52%)
Not interested in health overall. Feel they can take care of themselves. Unnecessary	59 (20%)	13 (13%)	12 (14%)	26 (17%)	110 (17%)
Convenience. DMHS may be a long distance away and time-consuming to get to	53 (18%)	18 (18%)	9 (11%)	19 (12%)	99 (16%)
Require more information about the service	23 (8%)	6 (6%)	2 (2%)	5 (3%)	36 (6%)
Do not see the need for a gender distinction	9 (3%)	4 (4%)	4 (5%)	4 (3%)	21 (3%)
Did not provide reason	18 (6%)	6 (6%)	4 (5%)	7 (5%)	35 (6%)

*YH* young healthy, *YS* young sick, *OH* older healthy, *OS* older sick

### Health behaviours: Help-seeking

**Fig. 2:**
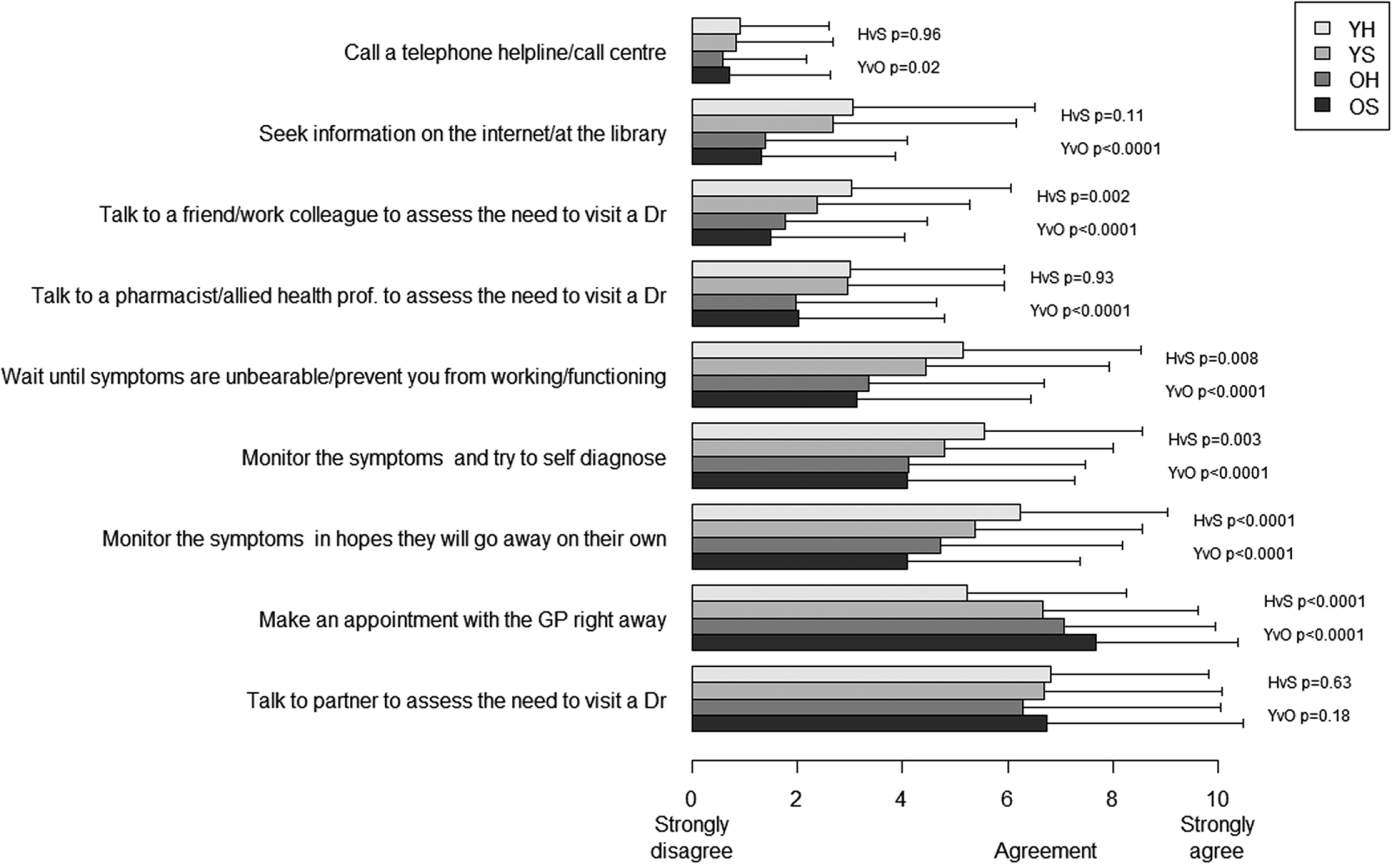
Help-seeking behaviours (mean ± SD) by age and health status

Older sick men were most likely to immediately make an appointment with a GP (mean ± SD = 7.7 ± 2.7) followed by older healthy men (7.1 ± 2.9), young sick men (6.7 ± 3.0) and young healthy men (5.2 ± 3.0) (F-test *p* < 0.0001) (Fig. 2). Conversely, younger men were much more likely to self-monitor symptoms in the hope of unaided recovery, try to self-diagnose, wait until unbearable symptoms, talk to others (friends, colleagues, pharmacists etc), or seek information online or at the library (all *p* < 0.001). Irrespectively of age, healthy men were more likely than sick men to monitor symptoms (*p* < 0.001), attempt self-diagnosis (*p* = 0.003), wait until unbearable symptoms (*p* = 0.008) and talk to a friend or colleague (*p* = 0.002). At least 50% of both older and younger men indicated high agreement (median = 8) that they would discuss the need to visit a doctor with their partners. No differences were observed with regards to health status for talking to a pharmacist or allied health professional, seeking information online or at the library, or calling a helpline (all *p* > 0.05).

Linear regressions of the two principal components for help-seeking behaviour (self-monitoring and information seeking) onto age (continuous) and health status (healthy v sick) indicate that young healthy men are more likely to self-monitor (*p* < 0.001) and seek information (*p* < 0.001) than older men, while sick men are less likely to self-monitor (*p* < 0.001) and slightly more likely to seek information (*p* = 0.06) than healthy men (Additional file 1: Table S4). No evidence of age by health status interactions were detected in either analysis (both *p* > 0.05).

### Health behaviours: Delay/avoidance

**Fig. 3:**
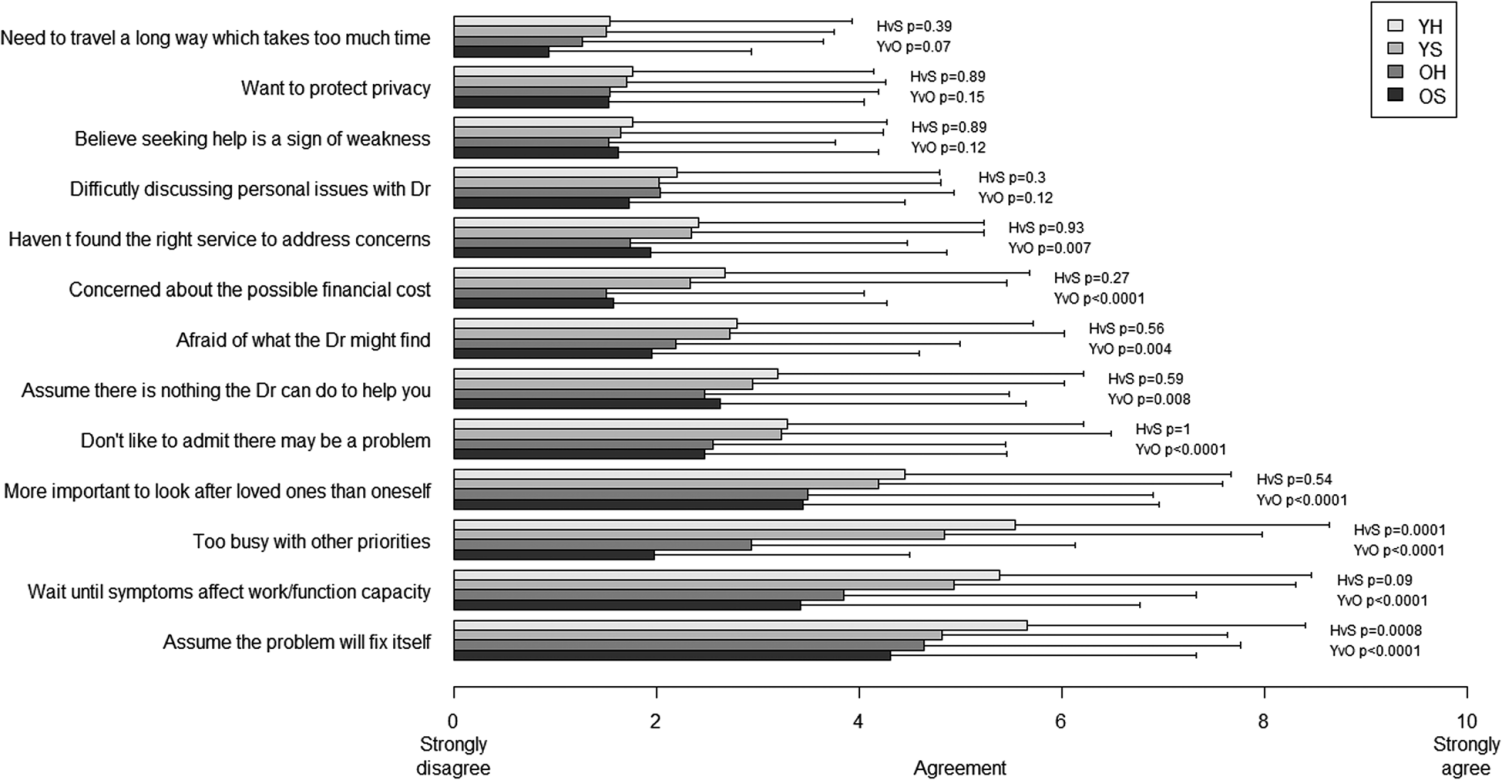
Agreement on statements of possible reasons for delay/avoidance behaviour (means ± SD) by age and health status

Sixty-eight percent of men (*n* = 1017) stated that they delay or avoid visiting a doctor or other health professional to address their health concerns at least some of the time, but this was more common in younger men (*p* < 0.001) and slightly more likely in healthy men (*p* = 0.01) (YH-78%, YS-73%, OH-59%, OS-45%). Of these men who reported delay/avoidance,  979 rated the reasons offered for doing so. In young healthy men the median agreement rating was at least 5 to the statements that they delayed or avoided visits to the doctor because they i) assumed that the problem will fix itself; ii) waited until symptoms affected their capacity to work or function; iii) were too busy with other priorities; and iv) considered it more important to look after their loved ones (Fig. 3). In older men, the only reason that received a median agreement rating of at least 5 was for the statement that they assumed that the problem will fix itself.

In the multivariable regression, men reporting a high delay/avoidance score were more likely to be those who self-monitored (*p* < 0.001) and were younger (*p* < 0.01). No associations were detected with either information-seeking (*p* = 0.17), nor health status (*p* = 0.37) (Additional file 1: Table S5).

### Factors influencing likelihood of attending a DMHS

**Fig. 4:**
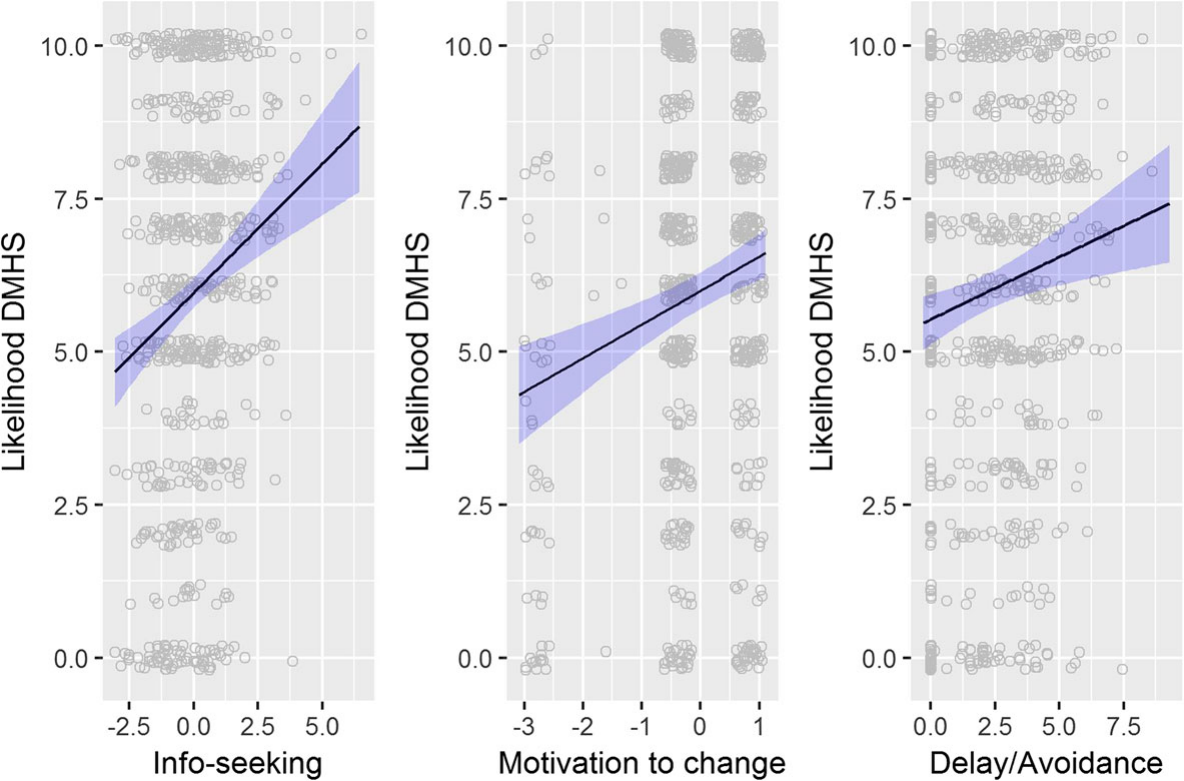
Multivariable associations between likelihood of attending a DHMS and info-seeking, motivation to change and delay/avoidance behaviours in young healthy men. Other covariates are set at their cohort means

In the multivariable regressions for all men, and for young healthy men only, information seeking men and men reporting high motivation to change their health reported a higher likelihood of attending a DMHS irrespective of age, health status or delay/avoidance behaviour status (all *p* ≤ 0.0001; Table 3, Additional file 1: Table S6, & Fig. 4). Men reporting health concerns (all *p* ≤ 0.02) and/or delay/avoidance behaviour (all *p* ≤ 0.02) were also associated with a greater likelihood of attending a DHMS. An interaction between age and health status was observed indicating that older sick men were less likely to report interest in attending a DMHS (*p* = 0.009), however this difference was attenuated in the men reporting delay/avoidance behaviour (*p* = 0.26). Although some of these associations were strongly significant, the total variation explained was low ranging from R^2^ = 6% to 9% across the four models.

**Table 3:** Linear regressions of likelihood of attending a DMHS onto the two help-seeking (self-monitoring and info-seeking) components, delay/avoidance behaviour, health concerns, motivation to change, weight-loss history, age and health status (healthy v sick)

Factors influencing likelihood of attending a dedicated men’s health service						
	All men (*N* = 1493)			Young healthy men (*N* = 733)		
	Est	SE	*p*-value	Est	SE	*p*-value
Intercept	5.5	0.2	< 0.0001	5.4	0.2	< 0.0001
Self-monitoring	−0.040	0.066	0.54	0.054	0.093	0.56
Info-seeking	0.37	0.06	< 0.0001	0.42	0.08	< 0.0001
Delay/Avoidance	0.21	0.05	< 0.0001	0.23	0.07	0.0006
Health concerns	0.22	0.09	0.009	0.27	0.11	0.01
Motivation to change	0.34	0.09	0.0001	0.55	0.12	< 0.0001
Weight loss attempted	0.11	0.09	0.19	0.02	0.11	0.85
Age	0.0012	0.0072	0.87	−0.0012	0.0098	0.91
Health (Sick v Healthy)	−0.14	0.19	0.46			
Age-Health interaction	−0.032	0.012	0.009

## Discussion

We have established from a telephone survey that 70% of men reported at least moderate likelihood of attending a DMHS, with 23% of men rating likelihood as very high. Health information seeking from sources other than their GP was strongly associated with the likelihood of men using a DHMS. Consistent with this, men with health concerns, and those who were motivated to change their health reported a greater likelihood of attending a DHMS. In men who reported delay/avoidance in regards to visiting their GP, the likelihood of attending a DHMS was increased. This was particularly the case for younger men who were more likely than older men to prioritise work and family commitments over a trip to the doctor.

The risk of selection bias was reduced by a participant response rate of 64%, considered good for phone based surveys. This good response, aided by the use of a pre-survey advance letter, also yielded good representativeness, according to population distributions for age and a number of other demographic parameters (Table 1). Aboriginal and Torres Strait Islander men were, however, under-represented (0.7%) and sociocultural differences may exist with regards to preferences for sex-specific services. Additionally, respondents were not asked to self-report gender, thereby limiting understanding of gender identity on likelihood of attending a DMHS. Furthermore, research respondents of phone surveys are inclined to answer positively. The reality therefore, is that not all of the 70% of men who suggested a moderate or higher likelihood, would attend a DMHS. A more realistic likelihood, calculated by discounting the “very high” and “high” likelihood proportions by 50% and 25% respectively, gives a figure of 45.5% for all men, marginally higher amongst younger men.

International evidence on the value of a DMHS is limited. From 2005 to 2008, the Scottish Government piloted a national program comprising sixteen community-based DMHSs called Well Men Service Pilots (WMS), attended by 3367 men [21, 22]. The objectives of the WMS were to engage men, provide opportunities for health assessment and screening and offer advice on health related behaviours and referrals, to enhance men’s health. Despite a positive response to the program overall, semi-structured interviews of participants [22] suggested that men showed ambivalence to the idea of male-specific services, stating that they would have used the Well Men Service even if it was not male-focused. This suggests that men may see value in primary care services for low acuity problems that are separated out from traditional GP services. Some men indicated they were happy to participate in health activities with females. Others did support the idea of male-specific services merely because women-only services exist.

We use the results of this study and particularly men’s responses regarding self-monitoring to challenge the commonly made assumption that men are disinterested in their health. We argue instead that men, generally, monitor their health status and make conscious decisions about when and how to seek help. Self-monitoring behaviour is influenced by previous illness experience, ability to maintain regular activities and everyday tasks, and perceptions of the severity of the health condition [7]. In this study, 91% of men surveyed reporting being motivated to change their health, and we have shown previously that men who self-monitor their health were aware of, and had a genuine interest in, their health and wellbeing [7]. This reinforces the need to optimise, and make more available, evidence-based information and self-monitoring tools to support these health behaviours by men, inside and outside of traditional health services, including the workplace. Such resources should not only provide appropriate health literacy for men, but also incorporate behavioral change strategies, provide links to relevant services, and offer triggers and incentives to visit their doctor for health checks [23–25]. mhealth and ehealth technologies, such as smartphones and wearables, have great potential to support self-monitoring preferences by men, but which bring with them unique challenges in terms of quality, uptake and sustained use [26].

Men delay seeking professional help when health symptoms occur [7, 27–30], and the participants in this survey were no different in terms of how they negotiate their health. Sixty-eight percent of men agreed strongly that they delay/avoid visiting a doctor at least some of the time. As reported in other studies [31, 32], work and family commitments were common reasons for delaying doctor’s visits, and expectedly, young healthy men rated these as reasons more strongly than young sick and older men. Not only were younger men more likely to self-monitor than older men, but those who scored higher on delay/avoidance behaviour, reported a higher likelihood of attending a DMHS. The implications of these findings are that GP services, regardless of whether they are a DMHS or not, should design and market their services towards these men in a way that uniquely addresses the common reasons for delay/avoidance. More suitable opening times, reasonable waiting times, availability of information and reading materials pertinent to men’s health and doctors with good awareness of men’s health issues and their presentation, were the attributes that specifically appealed to younger working men in this study. These results are to be reported in more detail elsewhere. Practitioners should also be suitably trained in styles of communication to better engage men in more meaningful discussion about their health [11, 33].

## Conclusions

From this largely representative survey of men, 45–70% of men report a moderate or greater likelihood of attending a DMHS. Actual feasibility would need to be established by a formal analysis of the potential strengths and weaknesses of such a service within the existing health services market place to ensure viability.

## Additional file

Additional file 1: **Table S1.** Disposition for 4900 randomly selected numbers used in sampling. Table S2 Rotation matrix for the first two components of the self-monitoring/info-seeking PCA analysis. Magnitudes greater than 0.3 are highlighted. Table S3 Demographics. Table S4 Multivariable associations with the info-seeking and self-monitoring principal components. Table S5 Multivariable associations with the total delay/avoidance score. Table S6 Linear regressions of likelihood of attending a DMHS in men reporting delays/avoidance in seeking health advice. (PDF 284 kb)

## Abbreviations

ANOVA: Analysis of variance
DMHS: Dedicated men’s health service
GP: General practitioner
OH: Older and healthy
OS: Older and sick
YH: Young and healthy
YS: Young and sick
