# Supplementary material for: Health behaviours of Australian men and the likelihood of attending a dedicated men’s health service
Source: BMC Public Health. 2018 Aug 30;18:1078. doi: 10.1186/s12889-018-5992-6 (PMC6117954; doi:10.1186/s12889-018-5992-6)
Supplement: Supplementary file 1 — Table S1. Disposition for 4900 randomly selected numbers used in sampling. Table S2 Rotation matrix for the first two components of the self-monitoring/info-seeking PCA analysis. Magnitudes greater than 0.3 are highlighted. Table S3 Demographics. Table S4 Multivariable associations with the info-seeking and self-monitoring principal components. Table S5 Multivariable associations with the total delay/avoidance score. Table S6 Linear regressions of likelihood of attending a DMHS in men reporting delays/avoidance in seeking health advice. (PDF 284 kb) [file 12889_2018_5992_MOESM1_ESM.pdf]

Supplementary Table S1: Disposition for 4900 randomly selected numbers used in sampling

	**N (%)**
Randomly selected numbers	4900
Telstra message/disconnected/fax modem number	797 (16)
No answer after 10x attempts	394 (8)
Business number or incorrect number	60 (1)
Household in listing twice	15 (<1)
Not in area	24 (<1)
No adult male/ not qualified	988 (20)
Potential respondent unavailable	38 (1)
Non-english speaking	92 (2)
Incapable, ill, deceased	148 (3)
Household refused	489 (10)
Eligible resident refused (345)/terminated interview (4)	349 (7)
Eligible resident participated in survey	1506 (31)

Table S2: Rotation matrix for the first two components of the self-monitoring/info-seeking PCA analysis. Magnitudes greater than 0.3 are highlighted.

		**Principal Component Loadings**	
**No.**	**Help Seeking Behaviour Statement**	**PC1 Self-monitoring**	**PC2 Info-seeking**
1.	Seek information about your symptoms / concerns on the internet or at the library	0.17	**0.39**
2.	Make an appointment with the GP right away	**-0.40**	0.24
3.	Wait until the symptoms become unbearable or prevent you from working or functioning and then make an appointment with the GP	**0.47**	-0.19
4.	Monitor the symptoms yourself for a while and try to self-diagnose	**0.49**	-0.04
5.	Monitor the symptoms yourself for a while in hopes they will go away on their own	**0.52**	-0.16
6.	Call a telephone helpline / call centre	0.12	**0.46**
7.	Talk to your partner/spouse/girlfriend to assess need to visit a Dr	0.04	**0.33**
8.	Talk to a friend or work colleague to assess the need to visit a Dr	0.20	**0.41**
9.	Talk to a pharmacist or other allied health professional to assess the need to visit a Dr	0.16	**0.48**

Supplementary Table S3: Demographics

	YH<br>N = 733	YS<br>N = 279	OH<br>N = 193	OS<br>N = 288	All<br>N = 1493
**Age**					
Median	46	55	69	73	56
(Range)	(19 – 65)	(19 - 65)	(57 - 95)	(54 - 92)	(19 - 95)
**Employment status**					
Employed (FT/PT)	669 (91%)	243 (87%)	35 (18%)	24 (8%)	971 (65%)
Not Working/Student/Other	64 (9%)	36 (13%)	1 (<1%)	3 (1%)	104 (7%)
Retired	0 (0%)	0 (0%)	157 (81%)	261 (91%)	418 (28%)
**Occupation (if employed)**					
White collar	411 (56%)	133 (48%)	25 (13%)	10 (3%)	579 (39%)
Blue collar	258 (35%)	109 (39%)	10 (5%)	14 (5%)	391 (26%)
Not reported		1 (<1%)			1 (<1%)
**Marital status**					
Divorced/Separated	36 (5%)	22 (8%)	14 (7%)	19 (7%)	91 (6%)
Married/Defacto	559 (76%)	221 (79%)	154 (80%)	231 (80%)	1165 (78%)
Never married	135 (18%)	34 (12%)	10 (5%)	10 (3%)	189 (13%)
Widowed	3 (<1%)	2 (<1%)	15 (8%)	28 (10%)	48 (3%)
**Household income**					
<$50,000	127 (17%)	72 (26%)	126 (65%)	206 (72%)	531 (36%)
$50,000-$100,000	320 (44%)	118 (42%)	40 (21%)	47 (16%)	525 (35%)
$100,000-$200,000	200 (27%)	68 (24%)	14 (7%)	13 (5%)	295 (20%)
>$200,000	42 (6%)	11 (4%)	1 (<1%)	1 (<1%)	55 (4%)
Not reported	44 (6%)	10 (4%)	12 (6%)	21 (7%)	87 (6%)
**Health status**					
Healthy	733 (100%)	0 (0%)	193 (100%)	0 (0%)	926 (62%)
Sick	0 (0%)	279 (100%)	0 (0%)	288 (100%)	567 (38%)

**Number of Dr visits (12 months)**					
Not at all	114 (16%)	0 (0%)	8 (4%)	0 (0%)	122 (8%)
Once or twice	313 (43%)	45 (16%)	52 (27%)	18 (6%)	428 (29%)
3 to 5 times	195 (27%)	92 (33%)	77 (40%)	64 (22%)	428 (29%)
5+ times	108 (15%)	138 (49%)	56 (29%)	202 (70%)	504 (34%)
Not reported	3 (<1%)	4 (1%)	0 (0%)	4 (1%)	11 (<1%)
**Concerns regarding current health**					
Nothing worries me	219 (30%)	50 (18%)	75 (39%)	82 (28%)	426 (29%)
Not sure	10 (1%)	4 (1%)	3 (2%)	2 (<1%)	19 (1%)
Concern raised	503 (69%)	225 (81%)	115 (60%)	203 (70%)	1046 (70%)
Not reported	1 (<1%)	0 (0%)	0 (0%)	1 (<1%)	2 (<1%)
**Motivation To change**					
Don't need to improve health or change lifestyle	31 (4%)	6 (2%)	12 (6%)	19 (7%)	68 (5%)
Don't care to improve health or change lifestyle	21 (3%)	14 (5%)	6 (3%)	4 (1%)	45 (3%)
Don't know / not sure	5 (<1%)	1 (<1%)	5 (3%)	3 (1%)	14 (<1%)
Motivated, but don't know what to do / where to go	62 (8%)	19 (7%)	7 (4%)	10 (3%)	98 (7%)
Motivated, but can't seem to take action	325 (44%)	126 (45%)	45 (23%)	67 (23%)	563 (38%)
Highly motivated	289 (39%)	113 (41%)	118 (61%)	185 (64%)	705 (47%)
**Weight loss previously attempted**					
No	305 (42%)	93 (33%)	100 (52%)	144 (50%)	642 (43%)
Thought about it but didn't take action	45 (6%)	17 (6%)	15 (8%)	13 (5%)	90 (6%)
Yes	383 (52%)	169 (61%)	78 (40%)	131 (45%)	761 (51%)

YH young healthy, YS young sick, OH older healthy, OS older sick

Supplementary Table S4: Multivariable associations with the info-seeking and self-monitoring principal components.

	**PC1: Self-monitoring**			**PC2: Info-seeking**		
	Est	SE	p-value	Est	SE	p-value
**Intercept**	2.11	0.134	<0.0001	0.699	0.124	<0.0001
**Age**	-0.036	0.00249	<0.0001	-0.0136	0.0023	<0.0001
**Health status**	-0.327	0.0821	<0.0001	0.143	0.0759	0.06

Supplementary Table S5: Multivariable associations with the total delay/avoidance score.

	**Est**	**SE**	**p-value**
**Intercept**	2.88	0.171	<0.0001
**Self-monitoring**	0.614	0.0303	<0.0001
**Info-seeking**	-0.0453	0.0329	0.17
**Age**	-0.0146	0.00315	<0.0001
**Health status**	-0.0855	0.0963	0.37

Supplementary Table S6: Linear regressions of likelihood of using a DMHS in men reporting delays/avoidance in seeking health advice.

Factors influencing likelihood of using a dedicated men's health service						
	All men (N=967)			Young healthy men (N=553)		
	Est	SE	p-value	Est	SE	p-value
**Intercept**	5.7	0.23	<0.0001	5.6	0.3	<0.0001
**Self-monitoring**	-0.061	0.084	0.47	0.086	0.11	0.43
**Info-seeking**	0.31	0.073	<0.0001	0.33	0.09	0.0003
**Delay/Avoidance**	0.18	0.066	0.006	0.2	0.087	0.02
**Health concerns**	0.24	0.099	0.02	0.29	0.12	0.02
**Motivated**	0.39	0.1	0.0001	0.58	0.13	<0.0001
**Weightloss attempted**	0.14	0.1	0.17	0.072	0.12	0.57
**Age**	-0.00083	0.0085	0.92	0.0035	0.011	0.75
**Health (Sick v Healthy)**	-0.096	0.24	0.69			
**Age-health interaction**	-0.018	0.016	0.26			
